# Long Road to Identifying Peritoneal Mesothelioma: An Elusive Diagnosis

**DOI:** 10.7759/cureus.85973

**Published:** 2025-06-13

**Authors:** Thejas Swaroop Konduru, Gourav Gourisaria, Goksu Ozen, Pradeep Karunakaran Thozhuthumparambil

**Affiliations:** 1 Acute Internal Medicine, Sandwell and West Birmingham Hospitals National Health Service (NHS) Trust, Birmingham, GBR

**Keywords:** ascitis, mediastinal lymph nodes, mesothelioma, peritoneal, peritoneal nodules, recurrent

## Abstract

Peritoneal mesothelioma is a rare and aggressive malignancy that often presents with non-specific abdominal symptoms such as ascites, leading to delayed diagnosis and treatment. It poses significant diagnostic challenges, especially in the absence of classical risk factors like asbestos exposure.

We report the case of a 60-year-old woman with a background of coeliac disease and no known asbestos exposure, who presented with progressive abdominal distension and ascites. Initial investigations, including imaging, paracentesis, cytology and liver biopsy, failed to reveal a definitive cause. Despite multiple evaluations and treating a presumptive diagnosis of sarcoidosis, her symptoms persisted. One year after the initial presentation, imaging revealed peritoneal nodularity and enhancement. Following biopsy and immunohistochemistry, a multidisciplinary team discussion led to the diagnosis of peritoneal mesothelioma. The patient was commenced on chemotherapy which showed clinical improvement.

This case underscores the importance of maintaining a high index of suspicion for rare malignancies such as peritoneal mesothelioma in patients with recurrent unexplained ascites, even in the absence of classical risk factors. Early multidisciplinary involvement and persistence in diagnostic evaluation are essential for timely diagnosis and management, which may improve patient outcomes in complex presentations.

## Introduction

Mesothelial cells are specialised cells lining the body’s cavities and internal organs. The peritoneum, which is a membrane lining the abdominal cavity, is one of the many membranes on the body on which these cells can be found. The mesothelial cells present on the peritoneum are prone to a rare and aggressive cancer known as peritoneal mesothelioma.

It is estimated that the incidence of this type of mesothelial cancer (also known as a ‘mesothelioma’) is approximately two per 1,000,000 people annually [1]. Although peritoneal mesothelioma accounts for only 15% of all mesothelioma cases, it poses significant challenges in both diagnosis and treatment [2]. Among the several more commonly observed types of mesotheliomas is pleural mesothelioma, for which asbestos exposure is a well-established risk factor, but its link to peritoneal mesothelioma is less clear; fewer than 50% of patients report prior asbestos exposure [3], and some cases are considered idiopathic.

Peritoneal mesothelioma can present as a localized abdominal disease; however, most cases exhibit diffuse involvement of the peritoneum. It may also extend into the pleural cavity, leading to pleural effusion. Metastasis (spreading of the cancer) to abdominal and pelvic lymph nodes is rare, and extra-abdominal metastases are exceedingly uncommon [4]. There are no specific or reliable tumour markers for the diagnosis of peritoneal mesothelioma. Although CA-125 (a tumour marker) is often elevated, it lacks specificity as it can be elevated in many other conditions like ovarian cancer and is primarily useful for monitoring disease recurrence or progression rather than for initial diagnosis [5].

## Case presentation

A 60-year-old woman with a background of coeliac disease, hypertension, gastro-oesophageal reflux disease (GORD) and osteoporosis presented to the emergency department. She reported progressive abdominal distension over the past five months, associated with back pain, orthopnoea and lower limb swelling. She also reported difficulty climbing stairs. There were no symptoms suggestive of hepatic, renal or cardiac disease. Her family history was significant for bowel cancer in her father and brother. The patient was a retired carer from a nursing home with no prior history of asbestos exposure. She was a non-smoker and did not consume alcohol.

Her vital signs were stable. On examination, tense ascites with decreased breath sounds at both lung bases and bilateral pitting pedal oedema to the ankles were found. A firm, non-tender submental lymph node was palpable. There were no stigmata of chronic liver disease. Initial laboratory investigations including liver function tests were within normal limits; details are presented in Table 1.

**Table 1:** Laboratory investigations INR: International Normalized Ratio; eGFR: estimated glomerular filtration rate; NT-proBNP: N-terminal pro-B-type natriuretic peptide; Ig: immunoglobulin; CA: cancer antigen; CCP: cyclic citrulinated peptide

Laboratory tests	Parameters	Value	Reference range
Full blood count	White blood cells (x10^9 ^/ L)	8	(4.00-11.00)
	Red blood cells (x10^12^/ L)	4.97	(4.5-6.00)
	Haemoglobin (g/L)	141	(125-180)
	Haematocrit (L/L)	0.41	(0.4-0.5)
	Neutrophils (x10^9 ^/ L)	4.51	(1.70-7.50)
	Lymphocytes (x10^9 ^/ L)	2.78	(1.00-4.00)
	Monocytes (x10^9 ^/ L)	0.51	(0.2-0.8)
	Basophils (x10^9 ^/ L)	0.11	(0.00-0.10)
	Eosinophils (x10^9 ^/ L)	0.07	(0.1-0.4)
	Platelets (x10^9 ^/ L)	409	(150-400)
Haematinics	Ferritin (ug/L)	77	(22-275)
	Vitamin B12 (ng/L)	280	(51-128)
	Folate (ug/L)	4.2	(3.1-20.0)
Coagulation screen	INR	0.96	(0.80-1.20)
Urea and electrolytes	Sodium (mmol/L)	142	(135-145)
	Potassium (mmol/L)	3.9	(3.5-5.5)
	Urea (mmol/L)	4.7	(2.5-7.8)
	eGFR (mL/min)	60	(>90)
	Creatinine (umol/L)	82	(59-104)
	Calcium (albumin adjusted) (mmol/L)	2.68	(2.2-2.6)
	Phosphate (mmol/L)	1.27	(0.8-1.5)
Cardiac markers	NT-proBNP (ng/L)	93	(<400)
Inflammatory markers	CRP (mg/L)	3	(<5)
Lipids	Total cholesterol (mmol/L)	4.9	(2.5-5)
	High-density lipoprotein (mmol/L)	1	(>1.2)
	Triglycerides (mmol/L)	1.5	(<2.3)
Endocrine	Thyroid-stimulating hormone (mIU/L)	8.31	(0.35-4.94)
	Free thyroxine (T4) (pmol/L)	10	(9-20)
	Parathyroid hormone (pmol/L)	21.4	(2-11.3)
Liver function tests	Bilirubin (umol/L)	7	(<21)
	Aspartate aminotransferase (U/L)	20	(<37)
	Alanine aminotransferase (U/L)	14	(<45)
	Alkaline phosphatase (U/L)	102	(20-130)
	Gamma-glutamyl transpeptidase (U/L)	19	(<45)
	Albumin (g/L)	40	(35-50)
Liver screen	Immunoglobulin A (g/L)	2.92	(0.8-4)
	Immunoglobulin G (g/L)	13.6	(5.3-16.5)
	Immunoglobulin M (g/L)	0.86	(0.5-2)
	Serum protein electrophoresis	Normal	
	Kappa/lambda light chain ratio	2.081	(0.26-1.65)
	Ceruloplasmin level (g/L)	0.36	(0.2-0.6)
	Alpha-1-antitrypsin level (g/L)	1.9	(0.9-2)
	Transglutaminase level (CU)	3.4	(<20)
	Enhanced liver fibrosis (ELF) test, blood	9.3	(<9.5)
Ascitic fluid analysis	Ascitic fluid albumin (g/L)	28	
	Total ascitic fluid protein (g/L)	42	
	Serum-ascites albumin gradient	12	(transudate if >11)
	Lactate dehydrogenase (iu/L)	126	(exudate if >255)
	Amylase (iu/L)	<30	(<101)
	Triglycerides (mmol/L)	0.2	(<1.2)
	White cells (X 10^6^/L)	33	(<250)
	Polymorphs (%)	70	
	Lymphocytes (%)	30	
	Red cells	62	
	Culture and mycobacterial culture	No growth	
	Cytology	Degenerated mesothelial cells, lymphocytes and macrophages. No malignant cells.	
Viral markers	Hepatitis A IgM antibody	Negative	
	Hepatitis B surface antigen, total hepatitis B core antibody	Negative	
	Hepatitis C antibody	Negative	
	Hepatitis E IgM and IgG antibody	Negative	
	Epstein-Barr virus IgM and IgG antibody	Negative	
	Cytomegalovirus IgM antibody	Negative	
	Cytomegalovirus IgG antibody	Positive	
Auto-immune screen	Antinuclear antibody	Negative	
	Extractable nuclear antigen	Negative	
	Anti-neutrophil cytoplasmic antibody	Negative	
	Anti-CCP antibody	Negative	
	Liver and kidney microsomal antibody	Negative	
	Smooth muscle IgG	Negative	
	Gastric parietal cell antibody	Negative	
	Mitochondrial IgG antibody	Negative	
Tumour markers	Alpha-fetoprotein (kU/L)	1	(1-7)
	CA - 125 (kU/L)	11	(<35)
	Carcinoembryonic antigen (ug/L)	2	(<5)
	CA - 19-9 (kU/L)	16	(<37)
	CA - 15-3 (kU/L)	26	(<31.9)
Pleural fluid analysis	Cytology	Atypical cells of uncertain significance	
Miscellaneous	Haemoglobin A1C (mmol/mol)	40	(20-41)
	Lactate dehydrogenase, blood (U/L)	117	(<225)
	Angiotensin-converting enzyme level, blood (u/L)	30	(12-82)
	Amylase, blood (U/L)	52	(<110)

**Figure 1:**
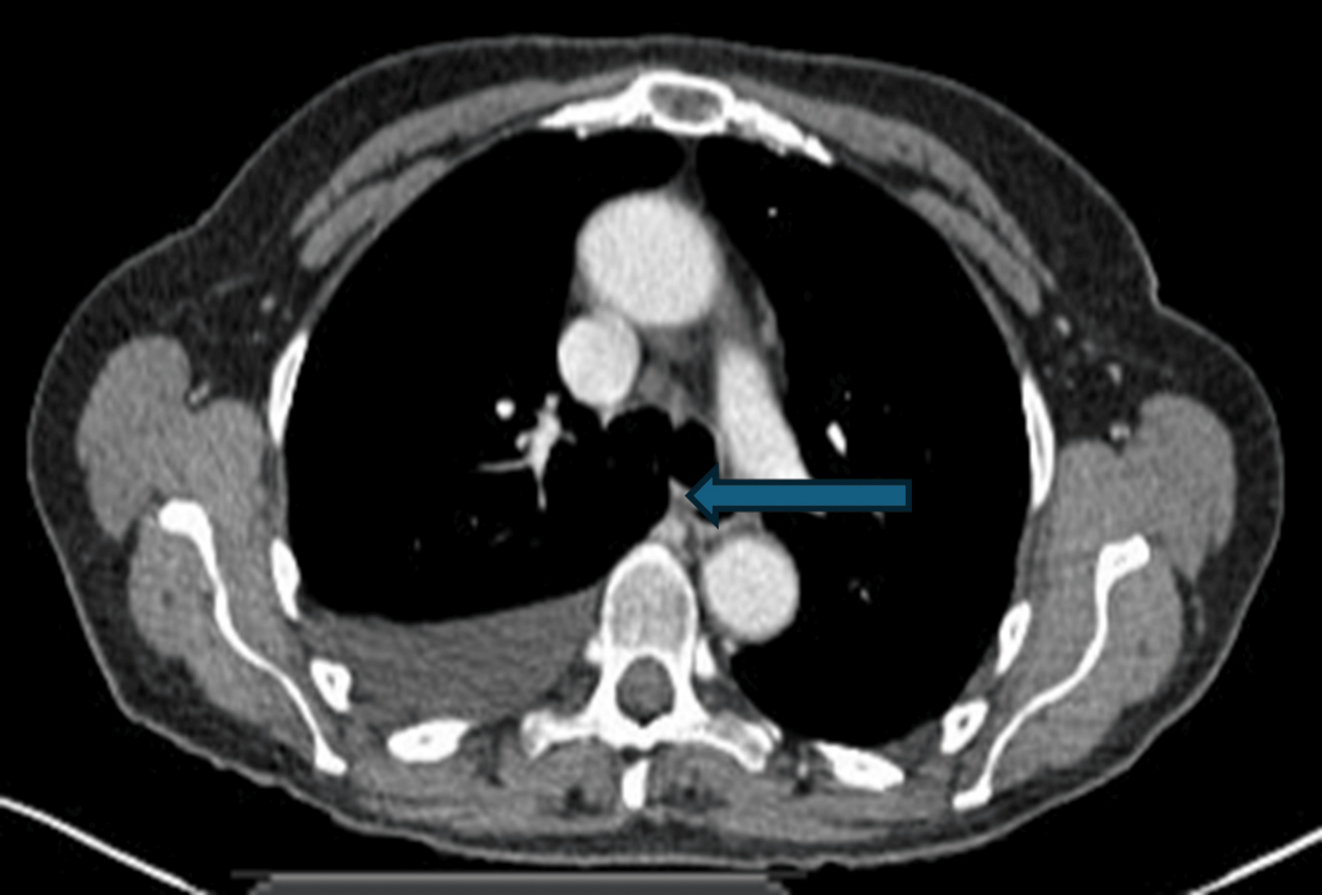
CT thorax axial section showing a pre-tracheal lymph node measuring 1.7 cm (blue arrow)

**Figure 2:**
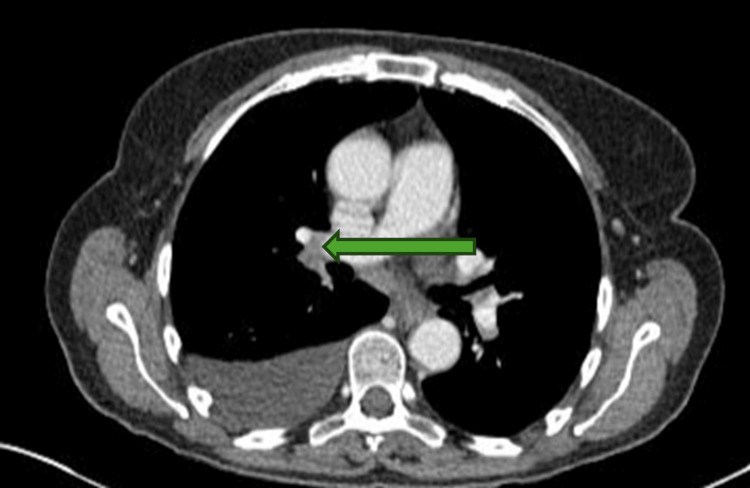
CT thorax axial section showing a hilar lymph node measuring 1.3 cm (green arrow)

As part of investigating the ascites, an ascitic tap was performed. The tap revealed a serum-ascites albumin gradient (SAAG) of 12, suggestive of transudative ascites (reference ranges provided in Table 1). She hence underwent a large-volume paracentesis (12 litres) with albumin replacement. Abdominal ultrasound showed normal hepatic echotexture and no evidence of obstruction. Subsequent computed tomography (CT) imaging confirmed these findings and additionally demonstrated a pre-tracheal lymph node measuring 1.7 cm (Figure 1) and a hilar lymph node measuring 1.3 cm (Figure 2), with mild bronchiectasis in the right middle lobe and a right-sided pleural effusion. The CT findings prompted a respiratory opinion, which advised imaging surveillance.

The above hepatic evaluations were inconclusive; hence further gastroenterology evaluation was performed. The tests conducted included viral hepatitis serologies, autoimmune panels and tumour markers, all of which returned negative results. She was initiated on spironolactone on account of the high SAAG ascites while awaiting ascitic fluid cytology. Given the lymphadenopathy, her serum kappa/lambda ratio was checked and found to be mildly elevated, but as her serum protein electrophoresis was normal and the absence of any laboratory findings supporting myeloma, it was considered insignificant.

The raised kappa/lambda ratio and lymphadenopathy, however, prompted a haematology review. This resulted in the recommendation of a neck ultrasound. This ultrasound identified a benign-appearing lymph node with preserved fatty hilum (which was hence not biopsiable as per the radiologist’s recommendation) and a coarse but normal-sized thyroid gland. Due to the coarse thyroid echotexture, we conducted a thyroid function test. The test revealed subclinical hypothyroidism, which was hence not pursued further. Ascitic fluid cytology revealed degenerated mesothelial cells, macrophages and lymphocytes but no malignant cells. The colonoscopy done as a result was normal. No cause for the ascites could be determined owing to the inconclusive test results, and the patient was discharged on spironolactone for the ascites.

Two months later, she was readmitted with worsening ascites. CT and ultrasound showed no significant interval changes. As a result, an enhanced liver fibrosis (ELF) test was conducted. The results indicated no significant hepatic fibrosis. Conservative management was pursued.

After five months, the patient was seen in the outpatient clinic and a follow-up CT scan was performed. The scan showed interval reduction in lymphadenopathy (hilar node was measured at 0.9 cm and pre-tracheal node at 1.4 cm), the reason for which was unclear. However, persistent right pleural effusion and large ascites were also observed. Owing to the recurring ascites and persistent pleural effusion despite spironolactone, a liver biopsy was conducted.

The liver biopsy revealed fatty changes but was otherwise non-diagnostic. Considering the persistent pleural effusion and lymphadenopathy, coupled with the inconclusive nature of prior investigations, an endobronchial ultrasound (EBUS) and lymph node biopsy of the mediastinal node was performed. The lymph node biopsy revealed non-necrotizing granulomatous lymphadenitis, which prompted evaluation for tuberculosis (TB). An interferon-gamma release assay (IGRA) and TB culture done as a result were negative. A presumptive diagnosis of indolent sarcoidosis was made keeping in mind the non-necrotising granuloma despite a normal serum ACE (Angiotensin-Converting Enzyme) level. A trial of oral prednisolone (5 mg) was initiated for the first six weeks as this was a case of long-standing, insidiously progressive disease. Despite the steroid, the patient continued to accumulate fluid, hence the prednisolone dose was escalated to 10 mg, which was continued for the next four months. Ascitic fluid continued to accumulate despite the above measures, at which point sarcoidosis was considered unlikely due to the lack of response despite four months of treatment and steroids were hence weaned off completely.

**Figure 3:**
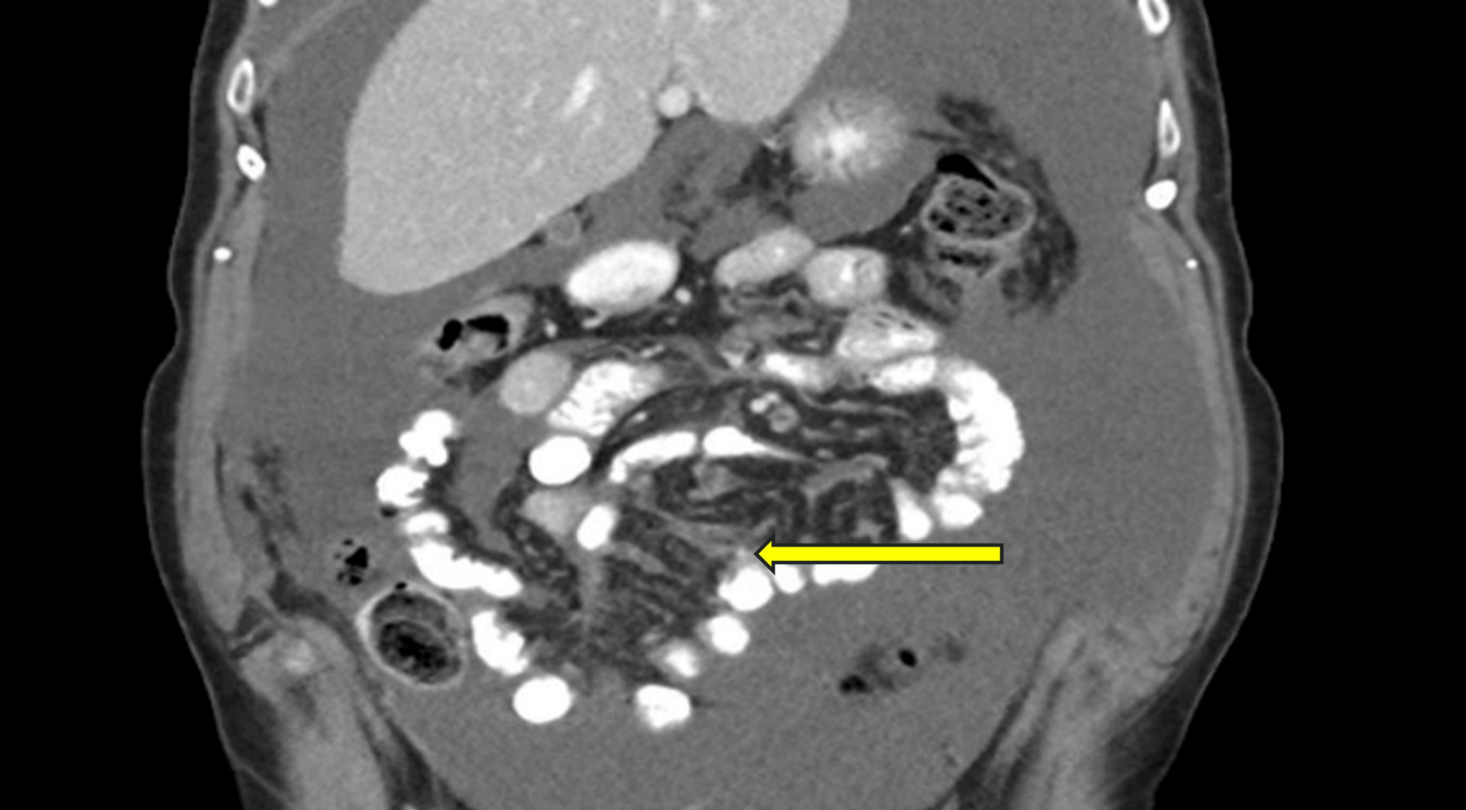
CT abdomen coronal section showing peritoneal nodules (yellow arrow)

One year later, a follow-up in the outpatient clinic was conducted. A CT scan conducted for routine surveillance revealed a gross increase in the size of the ascites, as well as pleural effusion and mild peritoneal wall enhancement with a few small peritoneal nodules (Figure 3), which were biopsied under ultrasound guidance.

Histology revealed very slender cores of tissue showing irregular angulated tubular structures embedded in a fibrotic stroma. A small focus showed a more complex architecture with marked branching. Whilst there was no definitive infiltration of fatty tissue, one of the tubular structures was noted in close proximity to fat tissue. Papillaroid fronds were noted in a couple of dilated ductal structures. On application of a panel of immunohistochemical stains, diffuse and strong staining was noted with Calretinin, CK5/6 and CK19. However, BerEP4 and MOC31 showed weak patchy staining. This immunoprofile did not allow a confident distinction between adenocarcinoma and mesothelial proliferation (atypical due to the architectural complexity), both of which remained in the differential diagnoses.

An upper gastrointestinal (GI) endoscopy was performed and found to be unremarkable apart from the presence of a hiatus hernia. Repeat analysis of ascitic fluid demonstrated an exudative pattern, with a SAAG of 4 in contrast to the initial presentation of transudative ascites. The reason for the shift was not entirely clear, but it was contemplated to be the evolving nature of the disease. Given the non-diagnostic histological and immunohistochemical findings, the case was reviewed at a multidisciplinary team (MDT) meeting. Subsequently, the histological slides were referred to a specialist pathologist with expertise in mesothelioma for further evaluation.

Additional immunohistochemical analysis revealed loss of nuclear expression of BRCA1-associated protein 1 (BAP-1), indicating a mutated status, while methylthioadenosine phosphorylase (MTAP) staining showed retained nuclear expression. The tumour cells were negative for epithelial markers including CK7, GATA3, CK20, CDX2, and TTF1. MOC31 and BerEP4 demonstrated equivocal (weak) staining. The morphological features and immunophenotypic profile were consistent with low-grade epithelioid mesothelioma of acinar pattern with BAP-1 mutation. In conjunction with radiological evidence of diffuse omental nodularity, these findings supported a final diagnosis of diffuse peritoneal mesothelioma of epithelioid type.

One month later, the patient developed worsening dyspnoea. CT imaging revealed a large right pleural effusion and 2 litres of fluid was drained. Cytology showed atypical cells insufficient for immunohistochemistry.

Her case was discussed in the mesothelioma MDT, and it was agreed upon that she was not a candidate for surgical intervention. She was referred for chemotherapy and was commenced on pemetrexed and cisplatin for peritoneal mesothelioma.

After starting chemotherapy, the patient subsequently underwent only one additional paracentesis for fluid re-accumulation. This marked a significant improvement considering that seven cycles of paracentesis were required previously.

## Discussion

Peritoneal mesothelioma is a rare malignancy originating from the serosal lining of the abdominal cavity, accounting for approximately 7%-10% of all mesothelioma cases [6,7]. Its diagnosis remains challenging due to its nonspecific clinical presentation, which often includes ascites, abdominal pain, weight loss, fatigue, and gastrointestinal symptoms. These features frequently mimic more common gastrointestinal or hepatic conditions, contributing to diagnostic delays.

In this case, the diagnostic process was further complicated by the presence of persistent mediastinal and pre-tracheal lymphadenopathy, which initially prompted extensive pulmonary evaluation and a presumptive diagnosis of indolent sarcoidosis. In addition, the persistently negative tumour markers and transudative nature of the ascetic fluid were misleading. Despite treatment, clinical improvement was not observed. This atypical feature significantly delayed the consideration of peritoneal mesothelioma, highlighting the importance of maintaining a broad differential diagnosis in cases of unexplained systemic symptoms and ascites. Notably, the presence of extra-abdominal lymphadenopathy should not exclude peritoneal malignancy from the diagnostic workup.

Imaging studies such as CT and ultrasound may reveal ascites, peritoneal thickening, or abdominal masses; however, these findings are not pathognomonic and may overlap with conditions such as peritoneal carcinomatosis or tuberculous peritonitis [8]. In this case, definitive diagnosis required tissue biopsy and immunohistochemical analysis. While immunohistochemistry is crucial, it may be inconclusive, particularly in the absence of a history of asbestos exposure, which is not universally present in peritoneal mesothelioma [9]. Loss of BAP1 and MTAP expression was instrumental in distinguishing malignant mesothelioma from benign mesothelial proliferations in this patient.

Histologically, peritoneal mesothelioma can present as epithelioid, sarcomatoid, or biphasic subtypes. These may further demonstrate variability based on cellular morphology, degree of stromal invasion, desmoplasia, and necrosis [10]. Differentiating primary peritoneal mesothelioma from secondary peritoneal carcinomatosis is essential and requires confirmation of mesothelial origin through immunohistochemical markers.

The peritoneal cancer index (PCI) is a widely used tool for staging and prognostication in peritoneal mesothelioma. It stratifies disease burden across 13 abdominal regions, with scores ranging from 0 to 39 [11]. Higher PCI scores are generally associated with poorer outcomes, especially when complete cytoreduction cannot be achieved [11].

First-line treatment typically includes pemetrexed in combination with a platinum-based agent, which has been shown to improve survival outcomes compared to cisplatin alone [6]. Immunotherapy is an emerging modality, with checkpoint inhibitors such as nivolumab (Opdivo) and ipilimumab (Yervoy) showing promise in recent trials [12].

The most effective intervention for select patients remains cytoreductive surgery (CRS) combined with hyperthermic intraperitoneal chemotherapy (HIPEC). In a multicenter study, patients undergoing CRS and HIPEC with perioperative chemotherapy achieved a median overall survival of 67 months [13]. Completeness of cytoreduction was strongly correlated with survival outcomes. Additionally, bidirectional chemotherapy approaches using intraperitoneal cisplatin and intravenous ifosfamide have been successfully employed in patients with renal impairment [14,15].

In our patient, these aggressive interventions were not pursued due to a performance status of one, emphasizing the importance of individualized treatment planning based on functional status and comorbidities. Early recognition and prompt histopathological evaluation remain critical in improving outcomes for patients with this rare but aggressive malignancy.

## Conclusions

This case underscores the diagnostic complexity and clinical unpredictability of peritoneal mesothelioma. Despite thorough investigations and consideration of a broad differential diagnosis, the final diagnosis was delayed by over a year from the initial presentation. This delay reflects the rarity of the condition and its nonspecific clinical features. The patient's persistent, unexplained transudative ascites occurring in the absence of hepatic, cardiac, or renal disease and with normal tumour markers prompted repeated imaging studies. The diagnostic direction was initially misled by the presence of extra-peritoneal lymphadenopathy, which steered clinical suspicion toward a thoracic pathology. Ultimately, subsequent imaging revealed new peritoneal pathology and the ascitic fluid evolved from transudate to exudate. Although the initial peritoneal biopsy did not yield a definitive diagnosis, ongoing investigations and multidisciplinary team discussions ultimately established the diagnosis.

This case highlights the critical importance of maintaining a high index of suspicion for rare malignancies such as peritoneal mesothelioma in patients with recurrent, unexplained ascites, especially when misleading features like extra-abdominal lymphadenopathy are present. A persistent, multidisciplinary approach to diagnostic evaluation is essential for timely diagnosis and optimal management, potentially improving outcomes in such complex clinical scenarios.
